# The complete chloroplast genome of *Clivia miniata*

**DOI:** 10.1080/23802359.2020.1721033

**Published:** 2020-02-03

**Authors:** Wei Wang, Fan Zhang, Chenhe Li, Xudan Zhou

**Affiliations:** College of Horticulture, Jilin Agricultural University, Changchun, China

**Keywords:** *Clivia miniata*, chloroplast genome, Amaryllidaceae

## Abstract

The complete chloroplast genome of *Clivia miniata* was assembled in this study. The genome comprised 158,114 bp in length. The GC content was 37.97%. A total of 133 genes are successfully annotated, including 87 protein-coding genes, 38 tRNA, and 8 rRNA genes. Seventeen protein-coding genes (*tpF, ndhA, ndhB, petB, petD, rpl16, rpl2, rps16, trnA-UGC, trnG-UCC, trnI-GAU, trnK-UUU, trnL-UAA, trnV-UAC, clpP, rps12, ycf3*) contained one or two introns. Phylogenetic tree analysis revealed that the *Clivia miniata* is the closest relative with *Lycoris radiata*, *Lycoris squamigera,* and *Narcissus poeticus*.

*Clivia miniata* belongs to *Clivia* of Amaryllidaceae. It originated in the deep forests of Natal Province, South Africa and was introduced to China in the early 20th century (Chen and Cheng 1990; Li 1988). *Clivia miniata* is widely used as a potted ornamental plant. It is also the city flower of Changchun City, Jilin province. At present, there is a great dispute about the phylogenetic position of the genus *Clivia miniata*, which intersects with Liliaceae and Asparagaceae. Amaryllidaceae and Liliaceae are closely related in system location. In taxonomy, different schools of thought hold different views on the boundary dividing the two families.

In this study, the complete chloroplast genome of *Clivia miniata* was sequenced and analyzed. Based on the complete chloroplast genome of *Clivia miniata* (GenBank accession number: MN857162), phylogenetic tree was constructed by maximum likelihood method using MEGA.7.0 to study the position of *Clivia miniata* in phylogenetic development (Kumar et al. 2016). The materials used in this experiment were taken from the horticulture laboratory of Jilin agricultural university (Changchun City, Jilin province, China, 43°48′37″N, 125°24′8″E). The specimens (NEFI20190802WW1) were kept in the horticulture laboratory of Jilin Agricultural University.

Pair-end Illumina raw reads were cleaned from adaptors and barcodes and then quality filtered using Trimmomatic (Bolger et al. 2014). All putative chloroplast reads mapped to the reference sequence above were then used for de novo assembly to reconstruct the chloroplast genomes using SPAdes 3.6.1 with iterative K-mer sizes of 55, 87, and 121 (Bankevich et al. 2012; Marcais and Kingsford 2011). De novo assembled chloroplast contigs were concatenated into larger contigs using Sequencher 5.3.2 Read coverage analysis was then conducted to determine the inverted repeat (IR) region boundaries and any misassembled contigs using Jellyfish v.2.2.3. Automatic annotation of the chloroplast genomes were generated by CpGAVAS and a circular representation of both sequences was drawn using the online tool OGDRAW. The draft annotations given by CpGAVAS were then manually corrected using the Artemis software and other plastid genomes for comparison (Liu et al. 2012).

The complete chloroplast genome of *Clivia miniata* is a typical four-segment structure with a total length of 158,114 bp, including a large single-copy region (LSC) with a length of 86,204 bp, a small single-copy region (SSC) with a length of 18,834 bp, and two reverse repeats of equal length but opposite direction (IRa/IRb) with a length of 26,788 bp. A total of 133 genes were encoded, including 87 protein-coding genes, 38 tRNA genes, and 8 rRNA genes with GC% of 37.97% and 36 duplicated genes in the IR region, which are *atpF, ndhA, ndhB petB, petD, rpl16, rpl2, rps16, trnA-UGC, trnG-UCC, trnI-GAU, trnK-UUU, trnL-UAA, trnV-UAC*, is contained in the introns, clpP and rps12 and ycf3 contains two introns.

Phylogenetic analysis was performed using the complete cp genomes of *Clivia miniata* with those of 10 species in Liliaceae,3 species of Amaryllidaceae, *Agave Americana,* and *Iris sanguinea* reported in Genbank of NCBI database by maximum likelihood method in MEGA version 7.0. The results showed that *Clivia miniata* and *Narcissus poeticus*, *Lycoris radiate*, *Lycoris squamigera* are in the same branch, belongs to Amaryllidaceae and is closely related to *Allium ampeloprasum* and *Allium obliquume* (Figure 1).

**Figure 1.**
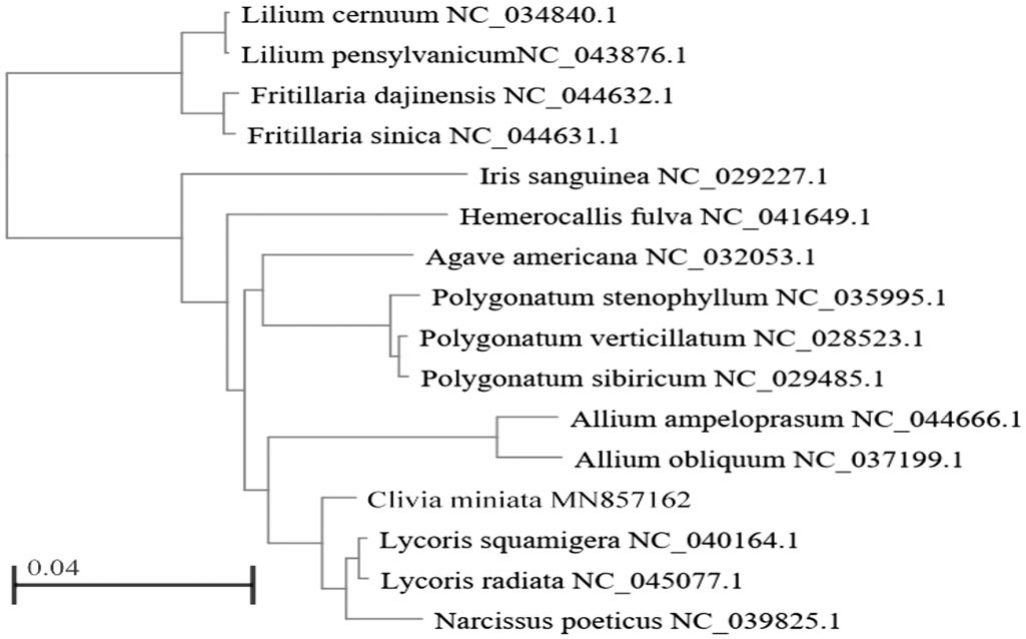
Phylogenetic tree based on chloroplast genome sequences from 16 plant species.

## References

[CIT0001] Bankevich A, Nurk S, Antipov D, Gurevich AA, Dvorkin M, Kulikov AS, Lesin VA, Nikolenko SI, Pham S, Prjibelski AD, et al. 2012. Spades: a new genome assembly algorithm and its applications to single-cell sequencing. J Computat Biol. 19(5):455–477. 10.1089/cmb.2012.0021 PMC3342519 22506599

[CIT0002] Bolger AM, Lohse M, Usadel B. 2014. Trimmomatic: a flexible trimmer for Illumina sequence data. Bioinformatics. 30(15):2114–2120. 24695404 10.1093/bioinformatics/btu170 PMC4103590

[CIT0003] Chen JY, Cheng XK. 1990. Chinese flower classics. Shanghai: Shanghai culture press.

[CIT0004] Kumar S, Stecher G, Tamura K. 2016. MEGA7: molecular evolutionary genetics analysis version 7.0 for bigger datasets. Mol Biol Evol. 33(7):1870–1874. 27004904 10.1093/molbev/msw054 PMC8210823

[CIT0006] Li ZQ. 1988. Chinese clivia. Chengdu: Sichuan Science and Technology Press. p. 20–47.

[CIT0007] Liu C, Shi LC, Zhu YJ, Chen HM, Zhang JH, Lin XH, Guan XJ. 2012. Cpgavas, an integrated web server for the annotation, visualization, analysis, and Genbank submission of completely sequenced chloroplast genome sequences. BMC Genomics. 13(1):715. 23256920 10.1186/1471-2164-13-715 PMC3543216

[CIT0008] Marcais G, Kingsford C. 2011. A fast, lock-free approach for efficient parallel counting of occurrences of k-mers. Bioinformatics. 27(6):764–770. 21217122 10.1093/bioinformatics/btr011 PMC3051319

